# A Systematic Review of Chest-Worn Sensors in Cardiac Assessment: Technologies, Advantages, and Limitations

**DOI:** 10.3390/s25196049

**Published:** 2025-10-01

**Authors:** Ana Machado, D. Filipa Ferreira, Simão Ferreira, Natália Almeida-Antunes, Paulo Carvalho, Pedro Melo, Nuno Rocha, Matilde A. Rodrigues

**Affiliations:** RISE-Health, Center for Translational Health and Medical Biotechnology Research (TBIO), ESS, Polytechnic of Porto, R. Dr. António Bernardino de Almeida, 400, 4200-072 Porto, Portugalpaulocarvalho@ess.ipp.pt (P.C.); pam@ess.ipp.pt (P.M.); nrocha@ess.ipp.pt (N.R.)

**Keywords:** wearable sensors, chest strap, cardiac monitoring, heart rate, heart rate variability

## Abstract

This study reviews the scientific use of chest-strap wearables, analyzing their advantages and limitations, following PRISMA guidelines. Eligible studies assessed chest-strap devices in adults and reported physiological outcomes such as heart rate, heart rate variability, R–R intervals, or electrocardiographic waveform morphology. Studies involving implanted devices, wrist-worn wearables, or lacking validation against reference standards were excluded. Searches were conducted in PubMed, Scopus, Web of Science, and ScienceDirect for studies published in the last 10 years. The quality of the studies was assessed using the Mixed Methods Appraisal Tool, and results were synthesized narratively. Thirty-two studies were included. The most frequently evaluated devices were the Polar H10 and Zephyr BioHarness 3.0, which showed strong correlations with electrocardiography at rest and during light-to-moderate activity. Reported limitations included motion artefacts, poor strap placement, sweating, and degradation of the skin–electrode interface. None of the devices had CE or FDA approval for clinical use, and most studies were conducted in controlled settings, limiting generalizability. Ergonomic concerns such as discomfort during prolonged wear and restricted mobility were also noted. Overall, chest-strap sensors showed good validity and were widely used in validation studies. However, technical refinements and large-scale field trials are needed for broader clinical and occupational application. This review is registered in PROSPERO and is part of the SIREN project.

## 1. Introduction

The rapid evolution of wearable technology has revolutionized the collection of physiological and behavioural data, enabling continuous, non-invasive monitoring across clinical, occupational, and sporting settings [1,2]. These devices support a range of applications, from optimizing sports performance to remote patient monitoring and enhancing Occupational Safety and Health (OSH) [1,3].

Wearable sensors can be positioned at different anatomical sites depending on the type of data intended to be captured [4,5]. There are rings, smartwatches, smart bras, and chest straps [6]. Wrist-worn devices, such as smartwatches, are widely used for general activity tracking but often lack the accuracy required for high-performance contexts [7]. Chest-worn band sensors, commonly known as chest straps, have gained prominence for their superior accuracy in cardiac monitoring, especially when compared to more widespread wrist-worn devices [8]. Additionally, sensors to monitor physiological features can be integrated into firefighters’ uniforms and personal protective equipment (PPE) [9]. Wearable devices can monitor electrocardiogram (ECG), photoplethysmography (PPG), electroencephalogram, and electromyogram signals, and measure parameters such as heart rate (HR), blood pressure, respiratory rate, tidal volume, body temperature, and peripheral oxygen saturation [6].

Body-worn sensors are one sort of wearable gadget that has gotten a lot of attention for their ability to analyze a person’s physical and mental state in real time. This makes them useful for everything from improving performance to keeping an eye on health [10]. The data collected by chest-strap wearables vary depending on the sensors they incorporate. These devices usually have technologies like electrocardiography, accelerometry, and strain sensors that can detect things like HR, heart rate variability (HRV), respiratory effort, and body movement very accurately [11,12]. Even if they have certain benefits, there are still problems with long-term comfort, power efficiency, and user acceptance [13]. Nonetheless, as a result of their increasing use in both commercial and research settings, a large number of models with varying sensor compositions, data formats, and usability features have been produced.

This study arises from the lack of systematic synthesis concerning the types of chest-worn cardiac sensors, the nature of the cardiovascular data they collect, and their respective advantages and limitations across application domains. Previous reviews on wearable technologies for cardiac monitoring have typically adopted a broad scope [14,15,16,17], encompassing multiple device types or specific clinical populations, and often provided descriptive rather than critical syntheses [18,19]. In contrast, the present review focuses exclusively on chest-worn sensors, enabling a more detailed evaluation of their technical and ergonomic characteristics; it synthesizes evidence across clinical, occupational, and sports contexts, offering a broader perspective on applicability. A comparative table can be seen in Appendix A. This review aims to fill that gap by critically evaluating chest-worn band sensors, with a particular focus on their use in clinical, sports, and ergonomic settings for cardiac monitoring.

## 2. Methodology

This systematic review was conducted according to the PRISMA Preferred Reporting Items for Systematic Reviews and Meta-Analysis guidelines. ChatGPT (OpenAI, GPT-5, March 2025 version) was used to support translation and language refinement of the manuscript. This review is registered in PROSPERO (registration number: CRD1046374).

### 2.1. Research Question and Eligibility Criteria

The research was based on the following questions: Which chest-worn band sensors are available for cardiac monitoring, what types of cardiac data do they collect, and what are their key strengths and limitations in different contexts?

Eligibility criteria were defined according to the PICO framework:

- Population (P): Studies involving individuals across clinical, sporting, or occupational settings who use chest-worn band sensors.
- Intervention (I): The use of chest-worn band sensors for cardiac monitoring.
- Comparison (C): Between chest-worn bands or a chest-worn band compared to the gold standard.
- Outcome (O): Measurement of cardiac physiological parameters and assessment of the advantages and limitations of chest-worn sensors in various contexts.

Studies were considered eligible for inclusion if they were original research articles, either observational or experimental, that investigated chest-worn band sensors (e.g., biometric chest straps or chest belts). Eligible studies were required to report on the types of sensors embedded in chest-strap devices and the corresponding physiological data related to cardiac monitoring, such as heart rate measured via ECG and blood pressure. Studies also qualified if they discussed technological, clinical, or practical advantages and/or limitations associated with the use of chest-strap devices. Only publications written in English and with open access were considered. Lastly, only research released in the previous 10 years was included to guarantee currency and relevance. Studies were grouped for synthesis according to the purpose of the device, i.e., whether it was employed as a gold standard or being validated.

Studies were excluded if they focused exclusively on wrist-worn devices, such as smartwatches or smartbands, unless chest-worn sensors were also independently assessed. Additionally, research involving non-wearable technologies or sensors located outside the chest region (e.g., head-mounted, finger-based, or implantable devices) was not considered eligible for this review.

### 2.2. Information Sources

Studies were initially identified through a search conducted in the following databases: PubMed, Scopus, Web of Science, and Science Direct. After this initial analysis, manual methods were used to identify studies, in particular, the backward citation technique, where the bibliographic references of the included studies were analyzed. Relevant review articles were also consulted. The search of these sources was conducted on 14 May 2025.

All data extracted from the included studies, including study characteristics, quality assessment, and synthesis tables, are fully presented within this article. No additional datasets, statistical code, or Appendix A and Appendix B are available beyond what is reported here.

### 2.3. Search Strategy

A comprehensive search strategy was designed for PubMed, Scopus, and Web of Science databases, incorporating a combination of keywords and MeSH terms. The complete search strategy is detailed in Table 1. For the ScienceDirect database, where a maximum of eight Boolean operators is allowed, the following query was used: (“Chest Based Sensor” OR “Chest Strap” OR “Wearable”) AND (“Heart Rate” OR “Heart Rate Variability” OR “Cardiac Stress” OR “Electrocardiogram”). Filters were applied to limit the search to studies published in the last ten years, in English and Portuguese, and also to include only peer-reviewed articles.

### 2.4. Selection Process

Two reviewers independently screened all records identified in the initial search. Disagreements between the reviewers were resolved through discussion, and a third reviewer was consulted if necessary. Each study was assessed for eligibility based on the inclusion and exclusion criteria. Automated tools such as Rayyan were used to facilitate the screening process and manage duplicates. The final list of eligible studies was determined after full-text review. Appendix B presents the table with the search strings used in each database and the corresponding results obtained.

### 2.5. Data Collection Process

Data were independently extracted by two reviewers from each included study. Data extraction involved identifying the sample, the study aim, the study type, the key aspects of the chest-worn band sensors used, the comparison between wearable or gold standard sensors, the application setting, the type of cardiac parameters collected, the main results, and key strengths and limitations. Any discrepancies between reviewers were resolved through discussion. Authors were not contacted for missing or unclear data; absent variables were recorded as “not reported”.

**Figure 1:**
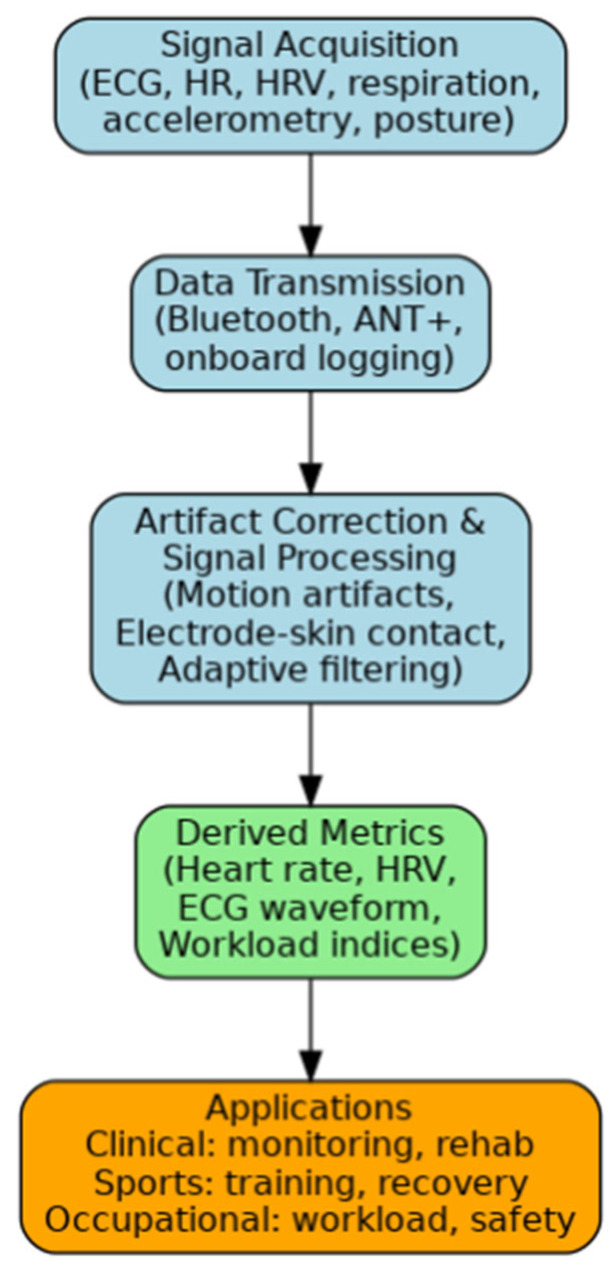
Conceptual framework of the functional pathway for chest-worn sensors.

Figure 1 represents the linear sequence from signal acquisition (e.g., ECG, HRV, or accelerometry) through data transmission and artefact correction, to the derivation of cardiac metrics and their eventual application in clinical, sports, or occupational settings. This framework highlights the interdependence between hardware design, signal processing algorithms, and end-user decision-making, reinforcing the need for integrated validation approaches.

### 2.6. Quality Assessment

This review’s studies were evaluated using the Mixed Methods Appraisal Tool (MMAT) from 2018 [20]. Two reviewers independently assessed study quality using the MMAT. Discrepancies were resolved by discussion. The MMAT is a recognized and validated method for appraising empirical studies with multiple methodological frameworks and boundaries. It allows evaluation of qualitative, quantitative (randomized, non-randomized, and descriptive), or mixed methods studies. Each study was categorized according to its study design (SD) as follows: SD1—qualitative studies; SD2—quantitative randomized controlled trials; SD3—quantitative non-randomized studies; SD4—quantitative descriptive studies; and SD5—mixed methods studies.

The MMAT comprises a series of standardized criteria tailored to each study design. S1 and S2, two preliminary screening questions, evaluate whether the research questions are well-articulated and whether the data gathered is sufficient to answer them. This is followed by five methodological quality criteria specific to each design. For example, when it comes to non-randomized quantitative studies (SD3), the tool looks at things like participant representativeness, measurement appropriateness, outcome data completeness, confounder control, and whether the intervention was carried out as planned. Every criterion has a rating of “Yes” (Y), “No” (N), or “Can’t tell” (CT). Studies were given an overall quality rating based on the quantity of criteria that were rated as “No” or “Can’t tell”: high quality—0 responses or 1 response of ‘No’ or ‘Can’t tell’; medium quality—2 responses of ‘No’ or ‘Can’t tell’; and low quality—3 or more responses of ‘No’ or ‘Can’t tell’. Although the MMAT authors advise against assigning overall ratings, this review reports them due to the identification of only two SD categories.

A strong interpretation of the body of evidence included in the review is supported by this systematic approach, which guarantees consistency and transparency in the evaluation of study quality.

The certainty of the evidence was not formally graded (e.g., using GRADE), as the review aimed to provide a descriptive overview rather than assess the strength of recommendations.

### 2.7. Effect Measures and Synthesis Methods

Effect measures included correlation coefficients, mean differences, and levels of agreement between chest-strap devices and reference standards, as reported in individual studies. Due to heterogeneity in study designs and outcomes, findings were synthesized narratively. Studies were tabulated by device type, validation context, and key outcomes. No statistical meta-analysis, heterogeneity analysis, or sensitivity analysis was conducted.

## 3. Results

**Figure 2:**
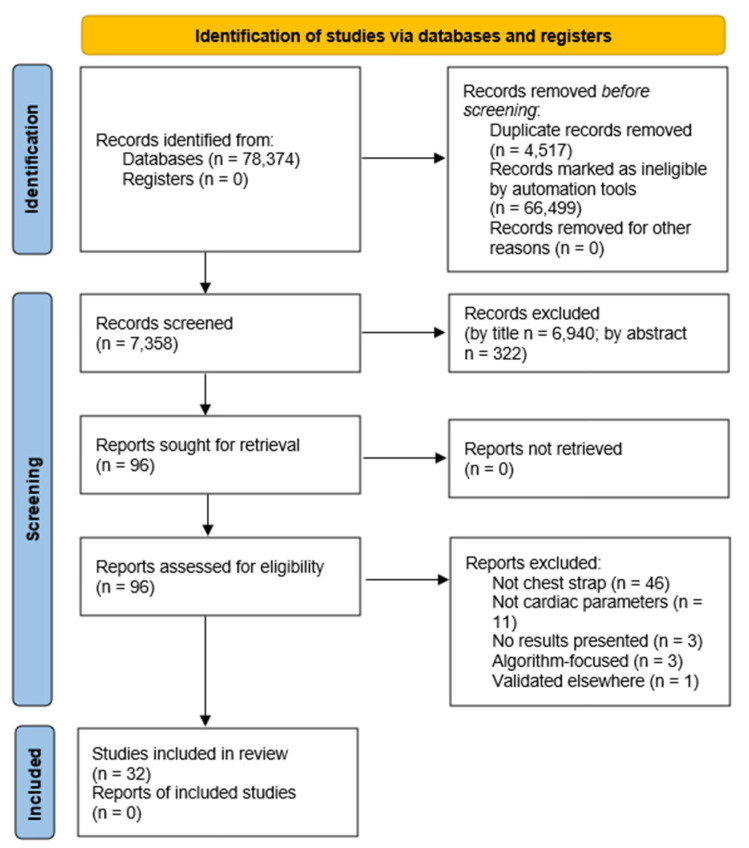
Flow diagram of study selection for this systematic review.

Figure 2 presents the flow diagram of study selection for this systematic review. The search strategies were applied to four databases, from which 78,374 records were identified: 9064 in PubMed, 13,318 in Web of Science, 21,288 in Scopus, and 34,704 in ScienceDirect. After applying filters (restricting the search to peer-reviewed articles published within the past ten years, written in either English or Portuguese), 11,875 records were obtained: 682 from PubMed, 4298 from Web of Science, 5926 from Scopus, and 969 from ScienceDirect. Using Rayyan, 4517 duplicate records were removed. With the same tool, 6940 records were excluded based on title analysis and 322 based on abstract analysis. Accordingly, 96 records were subjected to the eligibility criteria analysis. After full-text screening, 64 articles were excluded due to one of the following reasons:

1. Device type not aligned (not chest strap-focused): Studies were excluded under this category if they did not involve or focus on chest-strap-based ECG sensors. This included studies examining alternative devices such as patches, belts, wristbands, adhesive tapes, e-tattoos, or general electrode-based systems, as well as those that failed to specify the ECG device model used. Moreover, research that was only dedicated to the parts of a chest strap or comparing different methods of fixing a strap without considering the strap as a system was also excluded because it did not align with the review’s emphasis on holistic ECG monitoring based on chest straps.
2. Insufficient attention to cardiac parameters: Although the studies may involve physiological monitoring, the main concern remains focused on other parameters according to the various physiological components (energy expenditure/obesity, sleep/wake classification, training adaptation, or any other non-cardiac parameter). These articles were disqualified since the focus of this review is cardiac-specific metrics (such as heart rate, heart rate variability, and arrhythmias).
3. Data availability limitations (no results presented): Some references are methodological proposals, ongoing studies, or conceptual papers that lack experimental data or validation results. Others are guidelines or overviews without original data. These do not provide the empirical evidence required for the evaluation of sensor accuracy or applicability.
4. Algorithm-focused without sensor validation: A few studies concentrate on algorithm development or signal processing pipelines (e.g., arrhythmia detection or classification models), without actual validation of chest-strap sensor performance or without specifying the hardware used. Thus, they do not meet the inclusion criteria centred on device-level evaluation.
5. Duplicated or covered in other sources: Some studies use methodologies or datasets already validated and detailed in another included article, particularly when marked as “overnight” protocols, where relevant parameters were reported elsewhere.

Furthermore, many of the heart rate monitoring devices analyzed were patches or adhesive devices, which were not classified as chest straps and were therefore excluded from the review.

In this way, 32 studies were included in this review. To enhance clarity and analytical depth, the results of this review are presented in two separate tables, reflecting the distinct roles that chest-worn band sensors played across the included studies. This approach allows for a more targeted synthesis of the evidence and facilitates comparison within and across application contexts.

Table 2 includes studies in which chest-worn band sensors were the primary object of research. This table is divided into 2A (population, study type, aim, sensor, reference device, cardiac parameters measured, and application context) and 2B (reported results). These studies aimed to evaluate the performance of such sensors in terms of accuracy, usability, and applicability across different settings. They encompass four main types of research: (i) validation studies that assessed the sensor’s accuracy against a clinical gold standard (e.g., 12-lead ECG); (ii) comparative studies between different chest-worn sensors; (iii) application studies conducted in real-world environments (e.g., clinical, sporting, or occupational) without direct comparison; and (iv) studies focused on the technical development of new sensors or algorithms. This table highlights the key features, measured cardiac parameters, strengths, and limitations of each sensor in context.

Table 3, by contrast, includes studies in which chest-worn band sensors were not the primary focus but were instead used as a reference standard for validating other devices or technologies. These include smartwatches, wrist-worn PPG sensors, wearable patches, or novel data processing algorithms. In such studies, the chest-worn sensor served as the benchmark for assessing the accuracy of alternative cardiac monitoring tools. Although these studies do not evaluate the chest-worn sensors directly, their frequent use as a reference underscores their perceived reliability and methodological relevance.

By distinguishing between these two types of studies, this review aims to provide a more nuanced understanding of the role of chest-worn band sensors in cardiac monitoring. This structure also supports a more coherent discussion of their advantages, limitations, and application domains, whether as the focus of technological assessment or as a trusted reference in the validation of emerging solutions. Additionally, the inclusion of studies conducted in different settings (sports, clinical, and occupational) allows for covering the different requirements for a wearable applied to firefighters.

The analysis covered 32 articles, of which 68.75% were conducted in laboratory environments, 21.88% were conducted in real-world environments, and 9.38% combined both scenarios. In another analytical dimension, 6 articles focused on occupational users, 3 targeted athletes (professional or amateur), 7 were clinical (individuals with or without pathology), and 16 evaluated device performance in tasks with varying workload levels.

Table 2 shows that the most frequently used devices in scientific experimental contexts were Zephyr BioHarness 3.0 and Polar H10. Marzano-Felisatti et al. [28] used the Garmin HRM-Dual wearable in their experimental study. Di Palma et al. [33] chose to employ the Shimmer^®^ IFC-CNR chest strap in their feasibility study. Martín Gómez et al. [26], Parak et al. [34], and Rogers et al. [36] conducted their studies using the Movesense chest strap, in the HR+, Suunto, and Medical models, respectively. The Zephyr BioHarness 3.0 chest strap was the main tool used in the studies of Constantini et al. [22], Kuo et al. [27], Romagnoli et al. [37], Saggu et al. [38], Van Oost et al. [41], and Vila et al. [42]. Montes and Navalta [53] carried out their test–retest reliability study using the Polar T31 chest strap along with the Polar CE0537 wristband. Etiwy et al. [23], Mishra et al. [30], Nuske et al. [32], and Plews et al. [35] used the Polar H7 chest strap in their investigations. The Polar H10 chest strap was employed in the studies by Bläsing et al. [21], Flores et al. [24], Gilgen-Ammann, Schweizer, and Wyss [25], Maza, Goizueta, and Llorens [29], Skála et al. [54], and Speer et al. [40].

According to Table 3, the devices most frequently employed as reference technologies in research were Zephyr BioHarness 3.0, Polar H7, and Polar H10. Zephyr BioHarness 3.0 was adopted by Milena et al. [49], Kuo et al. [47], and Romano et al. [48] in their investigations. Higgins et al. [45], when evaluating an earpiece heart rate monitor, used the Polar T31 chest strap with the Polar FT1 wristband. Cosoli et al. [44], Liu et al. [48], and Navalta et al. [51] conducted their studies using the Polar H10 strap. In the studies by Chow and Yang [43], Hoevenaars et al. [46], and Navalta et al. [50], the Polar H7 strap was used.

Among the analyzed devices, those showing the highest performance in terms of HR measurement accuracy and precision were the Polar chest-strap models (H7 and H10). For example, Etiwy et al. [23] reported a correlation coefficient of 0.99 between Polar H7 data and reference values. Nuske et al. [32] found that Polar H7 achieved sample fidelity rates above 80% in high physiological variability contexts. Polar H10 demonstrated performance comparable to reference ECGs in both low- and high-intensity activities, even showing superior RR signal quality during vigorous movements [25]. Similarly, Bläsing et al. [21] found that the Polar H10’s signal precision was particularly relevant under high physical demand, with superior performance in phases of elevated HR.

Studies involving Movesense devices showed correlations above r = 0.95 in various HRV metrics [36], absolute mean errors below 1% (MAPE) [34], and residual means under 0.5 ms [26].

In clinical practice, Zephyr BioHarness 3.0 differentiated and detected clinically meaningful changes, such as arrhythmia and reduced HRV [37,38], and was also identified as the device to exhibit the best performance while changing exertion states [41].

Performance metrics when using wrist-worn devices, such as the Apple Watch and TomTom Spark, generally did not represent reliable data, especially when physically intense movement or exercise occurred. Wearable devices that are used in common everyday scenarios, such as Garmin, Fitbit, or Xiaomi, were used in contextually varied scenarios, and therefore, the applicability of performance measures was based on both exertion intensity and the target population. Chow and Yang [43] reported that the Garmin Vivosmart HR+ was found to be more accurate than the Xiaomi Mi Band 2 (although both manufacturers showed reduced accuracy as exercise intensity increased). Kuo et al. [47] and Romano et al. [52] assessed alternate technologies of video-based PPG and Inertial Measurement Unit (IMU) sensors, but they still posed significant issues of accelerated signal precision and stability, verified by the reported limits of agreement (LOAs), with findings suggesting Romano et al. [52] produced wide LOAs, especially in upright postures.

Ear sensors, as analyzed by Higgins et al. [45], showed a strong correlation with chest straps (r = 0.97), although methodological differences in heart rate calculation algorithms caused temporary discrepancies during sudden intensity changes. After analyzing the studies, the technical specifications of each of the chest straps were thoroughly compiled based on information from user manuals and official brand websites, and are presented in Table 4 [55,56,57,58,59].

In general, the devices do not include a screen or geolocation functionality, and all support real-time data transmission. To provide a structured synthesis, we consolidated the key advantages and limitations of chest-worn sensors reported across the included studies (Table 5). Chest straps consistently demonstrate superior signal accuracy compared to wrist-based devices, particularly in dynamic contexts, and are widely used as reference standards. However, limitations persist, including motion artefacts, strap discomfort, electrode–skin degradation, and the lack of regulatory approval for clinical use. By organizing findings into clinical, sports, occupational, and technical domains, this table supports a clearer evaluation of context-specific trade-offs and future opportunities for device development. This comparative overview highlights application-specific trade-offs, such as the balance between measurement accuracy and user comfort, or suitability for high-intensity versus low-intensity activities. Such a synthesis provides guidance for selecting the most appropriate device according to the research or monitoring context.

Regarding the quality assessment, two separate summary tables are presented, one for each type of included study. The methodological quality of the 22 studies listed in Table 2 is summarized in Table 6, while the 10 studies included in Table 3 are assessed in Table 7. All studies met the MMAT screening criteria concerning the clarity of research questions and the adequacy of data to address those questions. This review includes only two study types: the majority were quantitative non-randomized studies (SD3), while four were classified as quantitative descriptive studies (SD4).

Among the studies evaluating chest-strap devices, nine were rated as medium quality, eight as high, and three as low. All quantitative descriptive studies were rated high quality, although 75% of them failed to include participants representative of the target population. In the case of quantitative non-randomized studies (SD3), quality ratings were more variable. All medium- and low-quality studies fell within this category, while six studies were classified as high quality. All SD3 studies used appropriate measurements for both outcomes and interventions. However, several studies failed to adequately describe or address key methodological aspects, resulting in “Can’t tell” (CT) ratings for participant representativeness (27%), completeness of outcome data (5%), control of confounders (50%), and intervention fidelity (11%). Furthermore, some studies were rated “No” (N) in domains they did not consider methodologically, including participant representativeness (44%), completeness of outcome data (11%), and confounder accountability (22%).

Focusing specifically on studies using chest-strap devices as reference, all demonstrated appropriate measurements for both outcome and intervention, presented complete outcome data, and clearly explained whether the intervention/exposure occurred as intended. However, 60% failed to describe or address (CT) potential confounders, and 10% did not contemplate them at all (N). In terms of participant representativeness, 50% failed to consider this factor (N), and an additional 20% did not address it (CT). These gaps in reporting and methodological considerations limited confidence in the appraisal of certain studies.

## 4. Discussion

### 4.1. Overview of Chest-Strap Device Usage

The dominance of laboratory studies present in this review highlights a continued reliance on controlled environments to evaluate wearable cardiac monitoring systems for healthcare applications that may restrict the validity of findings, particularly for the usage of these wearables in uncontrolled environments [60]. Although real-world and mixed-context studies were limited in inclusion, the need for such studies is imperative to assess device robustness when implemented in meaningful, dynamic, and less controlled environmental conditions. For example, a number of recent studies have emphasized the importance of generating evidence in real-world conditions to address the lack of robustness [61]. Specifically, Keersmaeker et al. [62] developed a framework to unveil hidden implicit communication patterns that existed for smart home devices in less controlled conditions, and the study found 27% more unique flows due to the less controlled testing conditions than when utilizing the traditional means; Morales Casas et al. [63] conducted a semi-real validation of a medical device that had been installed within the information communication technology domain with emergency services, reporting a reliability outcome of 70–100% even though testing took place in difficult conditions; and Alkurdi et al. [64] transferred existing anxiety detection models that we constructed based on controlled conditions to real-world environments and reported that feature-based models performed better than traditional machine learning based on accuracy outcomes, despite the difference in noise conditions.

Furthermore, the concentration of studies around a few specific devices, particularly Polar H10, Zephyr BioHarness 3.0, and Polar H7, suggests a lack of diversity in technology evaluation, potentially driven by accessibility, brand trust, or legacy use [65,66]. Notably, although Polar models often outperform others in terms of accuracy and signal stability, especially under physical strain, devices like the Zephyr offer broader contextual usability, particularly in occupational and clinical domains [27,38,41,42,47]. This distinction reinforces the importance of considering not only technical performance but also situational adaptability when selecting reference technologies [67].

The overall analysis reveals that the most widely used devices across the different reviewed studies were the Zephyr BioHarness 3.0 and the Polar H10 chest straps. Their recurring selection as both primary tools and reference technologies suggests a level of popularity and perceived reliability that reinforces their status as gold standards in cardiac monitoring for scientific research.

The examination of the different contexts in which devices were used also demonstrates Zephyr BioHarness 3.0’s superior versatility in application compared to Polar H10. Specifically, Zephyr was employed in two sports studies [22,37], two experimental studies [49,52], four occupational studies [27,42,47,68], and one clinical study [38]; the results in all contexts were satisfactorily positive regarding its applicability. Polar H10, with the majority of its 11 studies noting the location, was primarily conducted in experimental contexts (5 studies), had lesser amounts of utilization in occupational (2 studies) and clinical contexts (2 studies), and was not performed in locations in the sports context in the reviewed articles. Based on this evidence, Polar H10 may be favoured in the current documentation; however, Zephyr BioHarness 3.0 will have more realistic, practical application potential in real-world, challenging contexts such as the occupation and sports contexts.

Chest-worn sensors generally outperform wrist-worn devices during physical activity because they measure the heart’s electrical signal (ECG waveform) directly at the thorax, close to the source, with stable electrode–skin contact [32,50,69]. In contrast, wrist-worn monitors rely on PPG, which indirectly infers heartbeats from peripheral blood volume changes and is highly vulnerable to motion artefacts, tissue perfusion variability, and optical interference [32,50,69]. Age-related changes can also influence the pulse shape detected by PPG, such as arterial stiffness, blood vessel dilation, fine wrinkles, loss of skin firmness, roughness, and mottled hyperpigmentation [43]. Validation studies consistently show that chest ECG devices, such as Polar H10, maintain accuracy across different exercise intensities, whereas wrist PPG devices display larger errors, particularly in beat-to-beat analyses and heart rate variability estimation [69,70,71]. Although recent advances in signal processing are improving wrist PPG performance for average heart rate monitoring, chest-worn ECG remains the reference method for reliable cardiac assessment in dynamic, real-world environments [69,70,71].

The inconsistent performance of wrist-worn and other wearables for general consumers reinforces the ongoing challenge to establish clinically validated accuracy in a more pleasing or commercially viable format [72,73]. While new methods such as video-based PPG and IMU-based systems show promise, there is a noticeable lack of validation for different postural and exertional contexts [74,75]. The ear-based monitoring technologies have potential, but their susceptibility to algorithmic inconsistencies, particularly upon sudden changes in activity intensity, also suggests the need for greater algorithm development [76,77]. Collectively, findings indicate the need for new validation processes centred on context, but also for the incorporation of end-user scenarios in research design.

### 4.2. Clinical Applications and Constraints

Cardiac rehabilitation programmes, arrhythmia screening, and remote physiological monitoring are promising application areas for chest-strap sensors [23,33,38,46,54]. In medical sciences, the 12-lead ECG remains the gold standard for detecting abnormalities [78]. While some studies included comparisons with ECG or Holter monitors [23,25,33,38,54], Di Palma et al. [33] had a small sample size, limiting generalizability to clinical populations.

Clinically, HRV is an increasingly important parameter for assessing autonomic balance, recovery, and cardiovascular prognosis [79,80]. Therefore, it is relevant in clinical practice that chest-strap wearables not only capture ECG waveforms (with high-quality morphology) but also HRV—something that was not achieved in any of the reviewed articles. Although non-invasive HR and HRV monitoring have shown increasing validity, reliability remains dependent on activity intensity, the technology used, study population, and application context [25,34,35,81]. Despite advances in connectivity, some sensors do not offer access to raw data or real-time extraction of HRV or ECG morphology, reducing their value in clinical contexts where detailed cardiac pattern analysis is essential [21].

In clinical applications, Etiwy et al. [23], Nuske et al. [32], Gilgen-Ammann et al. [25], Di Palma et al. [33], Saggu et al. [38], Skála et al. [54], and Speer et al. [40] used the Polar H7, Polar H10, and Zephyr BioHarness 3.0 sensors. However, these devices are not CE- or FDA-certified for clinical use and are usually sold as fitness wearables [21,82,83]. This lack of certification raises concerns regarding their use for diagnosis or clinical decision-making [21]. Although Zephyr BioHarness 3.0 is FDA-approved (510(k) Number: K113045) for the collection and transmission of ECG data, it is not intended for clinical use or direct diagnostic purposes.

Most validations were conducted at rest or during light exercise, which prevents extrapolation to high-intensity or high-stress scenarios [23,25,32,33]. Movement artefacts or improper strap placement can compromise data reliability, particularly in patients with limited mobility or excessive sweating [26,34,38].

### 4.3. Environmental and User Factors

The reviewed studies showed that external factors such as body movement, perspiration, environmental noise, and humidity significantly affect HR measurement quality, especially in chest straps with electrodes. Martín Gómez et al. [26] noted that sweat and moisture compromise the electrode–skin interface, affecting electrical conduction. Similarly, Nuske et al. [32] and Saggu et al. [38] reported that motion directly interferes with signal quality. Finally, Parak et al. [34] indicated that poor strap fit can introduce significant mechanical artefacts in the signal. These findings underscore the importance of testing devices under realistic usage conditions and considering environmental and user-related factors in sensor reliability assessments.

In occupational settings, environmental conditions can interfere with signal accuracy, particularly air pollution, environmental noise, body movement, and vibrations [84]. In high-risk environments, sensors display slow recovery times, limiting their capacity to respond instantly [85,86,87]. These constraints are consistent with the limitations identified in several of the included studies [28,32,34,42].

For wearable devices to be successful in workplace applications, they have to guarantee biocompatibility and skin permeability, as well as make it easy to use and remove, and monitor physically active workers during non-stop, dynamic daily activities, while maintaining signal quality free of errors and interference [6,88]. However, loss of skin contact due to sweating, in very physically active individuals, and wearing multiple layers of clothing represent important challenges that could impede ECG signal quality in some of the articles reviewed [26,34,38]. In extreme contexts, Cosoli et al. [44] explored devices’ performance during swimming, which presents an extreme challenge for physiological monitoring. Beyond environmental and technical considerations, user discomfort over prolonged wear remains a significant limitation, often rendering continuous use impractical [89]. More than half of the studies took place in laboratory environments where conditions are controlled.

### 4.4. Technical and Ergonomic Considerations and Limitations

From a technical perspective, functional robustness was highly valued, including real-time transmission [32], both online and offline analysis [33], high sampling rates [21], and long-lasting batteries [38]. Ergonomics and user comfort were also recurrent, especially regarding ease of use and fitting [21,38].

Overall, the findings from the included studies confirm the superiority of chest straps in contexts requiring high HR monitoring precision. As such, they remain the preferred choice in clinical research and applied settings where reliable physiological data is crucial.

Nonetheless, some common limitations should be noted, including small sample sizes, experimental protocol variability, and lack of data for submaximal or maximal intensities. These shortcomings point to the need for broader, longitudinal studies with standardized methodologies.

These observations are consistent with prior systematic reviews, which also reported high sampling rates and reliable data transmission as key technical requirements in wearable HR monitors [90,91]. Likewise, the ergonomic concerns identified in this review align with findings from broader evaluations of wearable usability across occupational contexts [89,90].

### 4.5. Quality Assessment

Regarding quantitative non-randomized studies, the predominance of medium-quality assessments highlights recurring methodological limitations—particularly concerning population representativeness and control of confounding factors. These limitations were often rated as “Can’t tell”, reflecting insufficient information for the evaluation. High-quality studies had no limitations for appropriate sampling strategies or analytic approaches that addressed confounding issues. Low-quality studies had poor clarity along the important domains of representativeness, confounder control, and completeness of outcomes. In fact, the findings highlight how poor reporting for non-randomized studies (with a physiological measurement device) detracts from credibility and transparency. This stands in contrast to the quantitative descriptive studies, all of which achieved high overall quality scores.

These findings are consistent with the previous literature, which has highlighted frequent limitations in non-randomized designs, particularly regarding inadequate control of confounding and incomplete reporting [91,92].

### 4.6. Main Methodological Limitations of the Review

The search was limited to four scientifically recognized databases for the identification of scientific articles. However, it is possible that relevant studies were not identified as the grey literature was not included. The results of this study are constrained by the fact that the identified and selected articles were restricted by the previously defined inclusion and exclusion criteria. Finally, heterogeneity in study designs, outcome measures, and reporting formats prevented the performance of quantitative synthesis, meaning that the conclusions are based exclusively on narrative synthesis.

## 5. Conclusions

This systematic review highlights that wearable cardiac monitoring devices, particularly chest-strap sensors, remain the benchmark technology due to their high accuracy, robustness, and adaptability across various application contexts. Among these, the Polar chest strap is the most widely used in laboratory settings, benefiting from broad availability and ease of use. However, the Zephyr chest strap has been identified in the literature as providing superior accuracy and reliability, especially under conditions involving intense physical exertion and significant movement.

Alongside chest straps, wrist-worn PPG devices are common, though they typically exhibit lower accuracy during motion compared to chest straps.

Chest-strap sensors demonstrate performance comparable to the clinical gold standard (ECG), maintaining reliability across clinical, sports, occupational, and educational settings. Their affordability and technical features, including high sampling rates, real-time data transmission, durability, and user comfort, support their integration into intelligent decision-support systems.

These devices are also frequently employed as reference standards in validating emerging technologies based on PPG, inertial sensors, and other novel modalities.

However, chest-strap sensors currently lack certain enhancements that could further improve their usability and functionality, such as extended data storage, multi-sensor integration, better adaptation to environmental variations, and improvements in user comfort related to fit and skin contact.

The findings of this review provide relevant guidance for multiple stakeholders. For healthcare professionals, chest-worn sensors may serve as valuable tools for supportive monitoring in rehabilitation, occupational health, and wellness programmes, although their current lack of CE/FDA approval limits diagnostic use. For researchers, the results highlight the need for standardized validation protocols, larger and more diverse clinical cohorts, and longitudinal field studies to improve comparability and strengthen translational evidence. For device manufacturers, the review identifies critical areas for innovation, including long-term comfort, electrode–skin stability, and motion artefact correction, which are essential for enhancing usability, reliability, and regulatory readiness. Together, these insights can inform future practice and accelerate the integration of chest-worn sensors into clinical, occupational, and sports applications.

Despite their clear potential, several gaps in the literature remain unresolved. Most existing studies rely on small cohorts of young, healthy participants, limiting generalizability to clinical or occupational populations. The scarcity of field-based investigations further restricts understanding of device performance under real-world conditions, where factors such as sweating, prolonged wear, and motion artefacts are most pronounced. In addition, heterogeneous methodologies and inconsistent reporting practices hinder cross-study comparability. To address these gaps, future research should consistently report key technical details—including sensor placement, calibration, and data quality checks—prioritize longitudinal and multicenter trials, and enroll more diverse and clinically relevant populations. Advances in signal processing for artefact correction and electrode–skin interface stability, together with stronger evidence to support regulatory approval, are urgently needed to fully define the role of chest-worn sensors in routine cardiac health assessment.

Overall, chest-strap sensors represent reliable, versatile, and scalable tools for accurate real-time cardiac monitoring, playing a key role in advancing scientific research, personalized clinical care, and remote monitoring in dynamic, demanding environments.

## Figures and Tables

**Table 1:** Query used in the systematic review.

“Chest Strap” AND	(“Chest Strap” OR “Thoracic Sensor” OR “Wearable Sensor” OR “Wearable devices” OR “Wearable” OR “Chest-worn” OR “Thoracic band” OR “Chest belt” OR “ Chest monitor”)
“Physiological Data”	(“Physiologic * Data” OR “Heart Rate” OR “HR” OR “Biometric” OR “Heart Rate Monitoring” OR “Biometry” OR “Blood Pressure” OR “Physiologic * signals” OR “Physiological measures” OR “Physiological Parameters” OR “Physiologic * Monitoring” OR “Ambulatory Monitoring” OR “Ambulatory Blood Pressure Monitoring” OR “Ambulatory Electrocardiography” OR “ECG” OR “Ambulatory Electrocardiography Monitoring” OR “Electrocardiogram” OR “Heart Rate Variability” OR “HRV” OR “Cardiac * stress”)

**Table 2:** Studies Evaluating Chest Straps. A—General description; B—Main results.

(A) Studies Evaluating Chest Straps. CPM—Cardiac Parameters Measured.							
Reference	Population	Study Type	Aim	Sensor (Model and Technology)	Comparison/ Reference Device	CPM	Application Context
Bläsing et al. [21]	13 healthy participants	Comparative experimental study	To compare the usability and data quality of various consumer and professional electrocardiogram (ECG) devices in both research and leisure contexts, by developing two novel approaches: one to assess local noise and waveform disturbances, and another to verify and classify RR intervals (RRIs).	Polar H10 (chest strap, 1000 Hz, Infrared) + Polar RS800 Multi (wrist/storage)	NeXus-10 MKII (chest patch electrodes, 8000 Hz, Bluetooth); eMotion Faros 360° (chest patch electrodes, 1000 Hz, Bluetooth); SOMNOtouch NIBP (chest patch electrodes, 512 Hz, Bluetooth); Hexoskin Hx1 (Shirt, 256 Hz, Bluetooth)	RRI; Heart Rate (HR)	Experimental: treadmill and leisure scenarios
Constantini et al. [22]	10 elite male distance runners	Experimental crossover study with within-subject comparison	To examine the effects of timing foot strikes to the systolic or diastolic phase of the cardiac cycle on heart rate, oxygen consumption, and ventilatory responses in elite distance runners.	Zephyr BioHarness 3.0 (chest strap)	Unpublished values and tolerances collected previously by authors JLB and PDM	HR; ECG waveform; RRI	Sports: treadmill
Etiwy et al. [23]	80 adults enrolled in a Phase II or III CR programme	Cross-sectional comparative validation	To assess the accuracy of four commercially available, optically based wearable heart rate monitors in patients with cardiovascular disease attending a cardiac rehabilitation programme at a tertiary care centre.	Polar H7 (chest strap)	Standard 12-Lead ECG (chest patch electrodes); 2 of these wristband HR monitors: Apple Watch, Fitbit Blaze, Garmin Forerunner 235, TomTom Spark Cardio	HR; ECG waveform	Clinical: treadmill
Flores et al. [24]	15 semi-professional soccer players	Cohort study	To analyze autonomic nervous system adaptations following musculoskeletal injury in athletes by measuring heart rate variability.	Polar H10 (chest strap, Bluetooth); Polar m200 (wristband/storage, Bluetooth)	No comparison device	Heart Rate Variability (HRV); RRI	Occupational (in lab)
Gilgen-Ammann, Schweizer, and Wyss [25]	10 healthy adults (5 male + 5 female)	Laboratory-based comparative validation study	To assess the RR interval signal quality of the medilog^®^ AR12plus Holter monitor and the Polar H10 chest strap at rest and during exercise in healthy individuals, using visual ECG inspection as the reference.	Polar H10 (chest strap, 1 ms)	Medilog^®^ AR12plus Holter (3-lead ECG Holter; chest patch electrodes, 1 ms, signals recovered)	RRI; ECG waveform	Experimental (in lab): sedentary activities, walking with workload, walking and running on treadmill
Martín Gómez et al. [26]	21 healthy adults	Experimental	To evaluate the validity and reliability of Movesense HR + ECG measurements across various exercise modes and intensities, using standard three-lead ECG as the reference, and to compare the performance of the Garmin HRM-Pro against the same criterion.	Movesense HR+ (chest strap, single-channel ECG, 500 Hz, Bluetooth)	ADInstruments (chest patch electrode, standard three-lead ECG, 1000 Hz) + Garmin HRM-Pro with Garmin Fenix 3 watch (chest strap + wristband)	R-R peak; ECG; HR; HRV	Experimental (in lab): treadmill or a cycle ergometer
Kuo et al. [27]	213 military aircrew trainees	Observational study, G tolerance prediction model development	To verify participants’ cardiac performance during walking using the CFI and to develop a formula predicting individual G tolerance in centrifuge training.	Zephyr BioHarness 3.0 (chest strap)	Omron 1100U sphygmomanometer (armband)	HR	Occupational
Marzano-Felisatti et al. [28]	30 physically active males	Experimental	To evaluate the accuracy of two chest straps and one armband during intermittent exercise in laboratory conditions, comparing their performance in effort and recovery phases to identify strengths and limitations of armband heart rate monitoring relative to chest straps.	Garmin HRM-Dual (chest strap, 4 Hz, ANT+); Coospo H6 (chest strap, 4 Hz, ANT+)	Coospo HW807 (armband, 4 Hz, ANT+)	HR	Experimental (in lab): cycle ergometer
Maza, Goizueta, and Llorens [29]	29 healthy participants	Validation	To investigate the reliability of a widely used low-cost chest strap in detecting HRV fluctuations in response to emotionally valenced stimuli, by assessing its similarity and agreement with a five-lead cardiac monitor under short-term and ultra-short-term conditions.	Polar H10 (chest strap, 1 Hz, Bluetooth)	Shimmer3 ECG (four-lead cardiac monitor, 8 Hz, Bluetooth)	HRV	Experimental: emotion recognition research
Mishra et al. [30]	27 university students	Validation and field study using commodity hardware	To evaluate the viability of using a commercially available heart rate monitor (Polar H7) to detect stress, by assessing its performance in both controlled laboratory settings and free-living conditions, as a low-cost alternative to clinical-grade sensors.	Polar H7 (chest strap; 1 Hz, Buetooth)	Biopac MP150 (standard ECG, chest patch electrode, signal recovered); Zephyr HXM (chest strap; 1 Hz, Bluetooth)	HR; HRV; RRI	Experimental (in lab/in loco): laboratory stress induction and real-life (field) monitoring
Montes and Navalta [31]	40 healthy young adults	Test–retest reliability study	To determine the reliability of the T31 heart rate monitor at rest and during motion-based activities, including free movement and treadmill exercise, in both male and female participants.	Polar T31 (chest strap) + Polar CE0537 (wrist/storage)	No direct comparison in this study	HR	Experimental (in lab); fitness, general exercise
Nuske et al. [32]	Study 1: 23 adults (typical); Study 2: 32 children with ASD and 23 typically developing children (8–12 yrs)	Two-phase feasibility and validation study (lab setting)	To evaluate the suitability, comfort, and validity of commercially available ambulatory cardiovascular monitors for measuring psychosocial stress in children with and without ASD, by first testing a validation framework in adults and then applying it to children.	Polar H7 (ECG, chest strap, Bluetooth)	Mio Fuse (PPG, wristband, Bluetooth); PulseOn (PPG, wristband, Bluetooth); Biopac MP-150 (standard ECG, chest patch electrode, signal recovered)	HR; HRV	Clinical: ASD stress assessment
Di Palma et al. [33]	5 male children with High-Functioning ASD; diagnosed with ADOS-2 and WISC-IV	Feasibility study (longitudinal, 6 months)	To assess autonomic nervous system responses in children with ASD during therapeutic sessions involving interactive serious games, using wearable technologies to correlate physiological signals with engagement levels and support therapy personalisation.	Shimmer^®^ IFC-CNR wireless ECG chest strap (chest strap, single lead, 200 Hz, Bluetooth)	ELA medical (Holter)	HR	Clinical: therapy with “serious games” for ASD
Parak et al. [34]	25 healthy adults	Validation of form factor for HR/HRV sensors	To compare the accuracy of a chest strap and a vest against a clinical ECG monitor for HR and HRV monitoring, and to analyze the impact of their accuracy on accumulated physiological metrics (Training Impulse (TRIMP), Excess Post-exercise Oxygen Consumption (EPOC), and energy expenditure (EE)) used in training monitoring and planning.	Suunto Movesense ECG (chest strap and sports vest, 125 Hz, Bluetooth)	Bittium Faros (3-lead Holter ECG, 256 Hz)	HR; HRV; RRI	Experimental (in lab): sports training and performance monitoring
Plews et al. [35]	26 healthy individuals (elite, well-trained, and recreational athletes)	Validation study during resting breathing (1 min)	To compare the accuracy and validity of HRV recordings obtained using a PPG smartphone application (HRV4Training) and the Polar H7 chest strap against the gold standard ECG.	HRV4Training smartphone app (photoplethysmography (PPG), video camera, 180 Hz); Polar H7 (chest strap, Bluetooth)	Cosmed Quark T12x (standard 12-lead ECG, chest patch electrode)	HRV	Sports: resting
Rogers et al. [36]	21 physically active adults	Cross-sectional validation study	To evaluate the agreement of the Movesense Medical chest-strap device (single-channel ECG) with a 12-channel ECG system for RR interval detection and selected HRV measures during rest, incremental cycling exercise, and post-exercise recovery.	Movesense Medical (chest strap; single-channel ECG; 512 Hz)	CardioPart 12 Blue (standard 12-lead ECG, 500 Hz)	RRI; HRV	Experimental: rest, ramp test, recovery
Romagnoli et al. [37]	51 healthy Caucasian athletes training 4 ± 1 times/week	Initial observational study for reference value development	To support large-scale prevention of sport-related sudden cardiac death by identifying electrocardiographic features that may serve as reference values in the pre-exercise phase.	Zephyr BioHarness 3.0 (chest strap, 1-lead ECG, 250 Hz, Bluetooth)	Reference values from clinical 12-lead ECG	HR; HRV; ECG waveform	Sports: pre-exercise monitoring
Saggu et al. [38]	34 patients [subgroup A: 20 inpatients (24 h), subgroup B: 14 ambulatory (12 weeks)]	Pilot observational feasibility study	To design and evaluate the feasibility of an investigational external cardiac monitor using a chest strap with single-lead dry electrodes for affordable long-term (3–6 months) cardiac monitoring, assessing its ECG diagnostic quality, patient comfort, and effectiveness in detecting cardiac arrhythmias compared to existing short- and long-term monitoring methods.	Zephyr BioHarness 3.0 + Reveal LINQ™ electronics (chest strap, single lead)	DR220 Holter (chest patch electrodes)	ECG waveform; HR; Inter-Beat Interval (IBI)	Clinical: short- and long-term monitoring
Skála et al. [39]	161 participants: hospitalized patients (54), outpatients (53), healthy controls (54)	Validation	To verify the feasibility of accurate long-term evaluation of all heartbeats on a single-lead ECG by an experienced cardiologist across patients with varying body types, rhythms, and cardiac devices, and to assess the presence of artefacts or noise that may hinder ECG evaluation in different patient groups.	Polar H10 (chest Strap, 1-lead ECG)	Standard 12-lead ECG (chest patch electrodes)	ECG waveform	Clinical: hospitalized and outpatient cardiology
Speer et al. [40]	146 healthy Australian preschool children (3–5 years old)	Cross-sectional study	To investigate the relationship between resting vagally mediated heart rate variability (HRV) and body mass index (BMI) in Australian preschool children aged 3 to 5 years.	Polar H10 (chest strap, Bluetooth 4.0)	Compared to electrocardiographic-derived recordings	HRV	Clinical: children’s relationship between resting vagally mediated HRV and BMI
Van Oost et al. [41]	24 healthy young adults (students)	Validation study in controlled dynamic protocol	To validate the accuracy of commercial wearable devices, including the Zephyr BioHarness 3.0 chest-strap device and six wrist-worn wearables, for heart rate measurement and stress monitoring in road freight drivers under both transient and steady-state conditions.	Zephyr BioHarness 3.0 (chest strap)	CAM-14 module (standard 12-lead ECG, chest patch electrodes)	HR	Occupational: drivers (in lab)
Vila et al. [42]	3 healthy male adults	Field validation and algorithm development	To develop and validate a signal quality index for data loss in IBI signals and assess the accuracy of a wrist-worn sensor against a wearable ECG in real-life conditions.	Zephyr BioHarness 3 (chest strap, 250 Hz, signal recovered)	Empatica E4 (wristband, 64 Hz, signal recovered); Pan-Tompkins ECG-derived IBI as academic reference	HR; IBI; HRV	Occupational (in loco)
(**B**) Reported results from studies evaluating chest straps.							
**Reference**		**Reported Results**					
Bläsing et al. [21]		Accuracy: Not reported (NR); Precision: NR. Device comparison: Polar has the highest percentage of missed beats during P3 (2-back) with 6.6% misclassification, but also the best result in P4 (0.02% misclassification). Polar scores better in phases with higher HR. Most scores were low or of insufficient quality (below 99%: 7 participants), mainly attributable to the 2-back task, but achieved very high scores during the running phase.					
Constantini et al. [22]		Accuracy: NR; Precision: NR. Device comparison: Group mean HR was significantly lower during diastolic compared with systolic stepping (*p* < 0.001); strong correlations were observed between diastolic and systolic stepping for HR, step rate (SR), and step length (*p* < 0.05, r = 0.95 for all comparisons)					
Etiwy et al. [23]		Accuracy: Polar chest strap (rc of 0.99). Among wrist-worn monitors, the Apple Watch performed best with rc = 0.80, followed by the Fitbit Blaze (rc = 0.78), TomTom Spark Cardio (rc =0.76) and Garmin Forerunner 235 (rc = 0.52); Precision: NR. Device comparison: Wrist-worn HR monitors, the Apple Watch, and TomTom Spark Cardio were most accurate, with no statistical difference from ECG (*p* = 0.62 for TomTom Spark Cardio and *p* = 0.09 for Apple Watch)					
Flores et al. [24]		Accuracy: NR; Precision: NR. Device comparison: Results show differences between T1 and T2 (*p* ≤ 0.05) in low-frequency power (n.u.) (*p* = 0.001) and high-frequency power (n.u.) (*p* = 0.001), in low-frequency/high-frequency ratio (*p* = 0.001), and in high-frequency power (ms2) (*p* = 0.017) measures. No statistical differences were found in low-frequency power (ms2) (*p* = 0.233). The low-frequency power (n.u.) was significantly lower after injury compared with LF power (n.u.) values after full return to play. In high-frequency power, there was a significant difference between the two moments with high values after injury.					
Gilgen-Ammann, Schweizer, and Wyss [25]		Accuracy: NR; Precision: NR. Device comparison: RR interval signal qualities of 94.6% and 99.6% were demonstrated for the medilog^®^ AR12plus and the Polar H10. During the high-intensity activities, the RR interval signal quality of the medilog^®^ AR12plus dropped to 89.8%, whereas the Polar H10 maintained a signal quality of 99.4%. The correlation between both systems was high (r = 0.997, *p* > 0.001).					
Martín Gómez et al. [26]		Accuracy: Movesense HR+ mean absolute percentage error (MAPE)= 1%; rC (Lin)= 0,99; Garmin HRM MAPE= 13%; rC (Lin)= 0,32; Precision: Movesense HR+ = 99.6%; Garmin HRM = 87.7%. Device comparison: Bland–Altman analysis compared to the criterion indicated mean differences (SD) in RR’ intervals of 0.23 (22.3) ms for Movesense HR+ at rest and 0.38 (18.7) ms during the incremental test. The mean difference for Garmin HRM-Pro at rest was −8.5 (111.5) ms and 27.7 (128.7) ms for the incremental test. The incremental test correlation was very strong (r = 0.98) between Movesense HR+ and the criterion, and moderate (r = 0.66) for Garmin HRM-Pro.					
Kuo et al. [27]		Accuracy: NR; Precision: NR. Device comparison: Walking cardiac force index (WCFI) positively correlated with Relaxed G tolerance (RGT) (r = 0.234; *p* = 0.001) and straining G tolerance (SGT) (r = 0.256; *p* < 0.001). RGT = 0.066 × age + 0.043 × (WCFI × 100) − 0.037 × height + 0.015 × SBP − 0.010 × HR + 7.724. SGT = 0.103 × (WCFI × 100) − 0.069 × height + 0.018 × SBP + 15.899.					
Marzano-Felisatti et al. [28]		Accuracy: NR; Precision: NR. Device comparison: The ICC (intraclass correlation coefficients) values indicate a strong agreement between the Garmin and Coospo chest straps (ICC = 0.6–1.0). However, lower ICC values between the Coospo Armband and both chest straps (ICC = 0.10–0.77) reflect the higher measurement of discrepancies, particularly during effort stages					
Maza, Goizueta, and Llorens [29]		Accuracy: NR; Precision: NR. Device comparison: Signals recorded by both devices were highly correlated with no significant discrepancies between measures; strong to excellent agreement in time-, frequency-, and nonlinear measures					
Mishra et al. [30]		Accuracy: NR; Precision: NR. Device comparison: F1-score up to 0.87 (lab) and 0.66 (field); strong correlation with clinical ECG (r > 0.95 for most features)					
Montes and Navalta [31]		Accuracy: NR; Precision: NR. Device comparison: Cronbach’s α from 0.90 to 0.99 across all test conditions; all *p* < 0.001; excellent reliability					
Nuske et al. [32]		Accuracy: NR; Precision: NR. Device comparison: HR ↑ and HRV ↓ during stress vs. rest (*p* < 0.001 for both devices); Sampling Fidelity ≥ 83%; Spike Rate ≤ 13%; η^2^ > 0.25 for HR and 0.16–0.26 for HRV effects					
Di Palma et al. [33]		Accuracy: NR; Precision: NR. Device comparison: Physiological events (↑ HR, ↓ Root Mean Square of Successive Differences (RMSSD), ↓ Respiratory Sinus Arrhythmia (RSA)) correlated with sociocognitive engagement; increased “lower RSA” and “lower RMSSD” events over time; ECG well-tolerated throughout					
Parak et al. [34]		Accuracy: Strap = 99,24%; Vest = 84,70%. Precision: NR. Device comparison: Chest strap: HR MAPE = 0.76%, EPOC MAPE = 3.90%, TRIMP MAPE = 0.38%; Vest: HR MAPE = 3.32%, EPOC MAPE = 54.15%, TRIMP MAPE = 8.99%; chest strap more accurate across all measures					
Plews et al. [35]		Accuracy: NR; Precision: NR. Device comparison: All differences vs. ECG were “trivial”; technical error of estimate (TEE) coefficient variation (CV) %: PPG GB = 3.8%, Polar H7 NB = 8.6%; correlations r = 0.99–1.00; mean bias < 2.0 ms					
Rogers et al. [36]		Accuracy: NR; Precision: NR. Device comparison: High correlations for HRV parameters: Pearson’s r = 0.95–1.00; small bias (e.g., meanRR PRE bias = 0.0 ms, Limits of Agreement (LOAs) ± 1.9 ms); short-term scaling exponent of Detrended Fluctuation Analysis (DFA a1) agreement r ≥ 0.95					
Romagnoli et al. [37]		Accuracy: NR; Precision: NR. Device comparison: Median values reported with interquartile range; significant differences found vs. clinical ECG norms (e.g., ↓ HRV, ↑ QRS duration, ↓ QT interval)					
Saggu et al. [38]		Accuracy: NR; Precision: NR. Device comparison: Diagnostic-quality ECG for 76.5% of monitoring; arrhythmia yield: 24% (24 h) and 64% (12 weeks); comfort reported in 94.9%					
Skála et al. [39]		Accuracy: NR; Precision: NR. Device comparison: Basic rhythm reliably determined in the majority of patients; 2.16% noise					
Speer et al. [40]		Accuracy: NR; Precision: NR. Device comparison: Significant inverse relationship between RMSSD (ln) and BMI (β = −0.06; 95% CI = −0.12–−0.01; *p* = 0.032)					
Van Oost et al. [41]		Accuracy: NR; Precision: NR. Device comparison: Zephyr showed near-perfect accuracy (MAPE and CCC) in dynamic HR; wrist-wearables varied: Fitbits performed best, WHOOP and Withings worst; transitions (HR dynamics) caused a performance drop in all devices					
Vila et al. [42]		Accuracy: NR; Precision: NR. Device comparison: Median error for mean HR: 3.2%; RMSSD: 62%; Low Frequency (LF): 25%; High Frequency (HF): 63%. Accuracy improved when no missing samples (0.0%, 27%, and 6.4%, respectively)					

**Table 3:** Studies using the chest-strap device as a reference tool. CPM—cardiac parameters measured.

Reference	Aim	Population	Sensor (Model and Technology)	Target Device	Reported Results (Reference vs. Target)	CPM	Application Context
Chow and Yang [43]	To compare the real-time heart rate (HR) tracking performance of two commercial fitness wearables (photoplethysmography (PPG)-based) in younger versus older adults during moderate physical activity	20 adults aged 65 years and above (Senior) and 20 adults aged between 20 years and 26 years (Young)	Polar H7 (chest strap)	Xiaomi Mi Band 2 (Xiaomi Corporation) and Garmin Vivosmart HR+ (Garmin International Inc)	The Garmin device produced more reliable and accurate HR readings than the Xiaomi one. The accuracy levels of both devices were negatively correlated with the level of activity intensity. For both devices, the measurement accuracy deteriorated in individuals while cycling.	HR	Experimental (in lab): treadmill, upright stationary bike, and elliptical machine: aerobic training.
Cosoli et al. [44]	To evaluate the accuracy and precision of wrist-worn (Polar Vantage V2, Garmin Venu Sq) versus chest-strap (Polar H10) HR monitors during swimming and dry-land activities in expert swimmers.	10 expert swimmers	Polar H10 (cardiac belt, 130 Hz)	Polar Vantage V2; Garmin Venu Sq	Precision and accuracy worsen in water tests. The metrological performance in terms of accuracy of Polar Vantage V2 is better compared to Garmin Venu Sq.	HR	Occupational/sports/experimental: swimming in different styles (in loco); walking/running on a treadmill (in lab)
Higgins et al. [45]	To evaluate the validity of an earpiece HR monitoring device against a previously validated chest-strap HR monitoring device	15 college students	Polar T31 (chest strap) + Polar FT1 HR monitor (Bluetooth)	Bioconnected wireless exercise earpieces	Device Correlation: Strong overall correlation between earpiece and chest strap (r = 0.97); Meets validity threshold (r ≥ 0.90) for HR monitoring devices. Measurement Accuracy: 521 ± 117 HR data points (earpiece) vs. 517 ± 118 (chest strap). Close overlap in readings for first 350 s of protocol. Max discrepancy: <10 beats per minute (bpm) during walk-to-jog transition. Algorithm Differences: Chest strap showed sudden HR spikes (5 sec averaging); earpiece demonstrated gradual increases (continuous monitoring).	HR	Experimental (in lab)
Hoevenaars et al. [46]	To assess the reliability of Fitbit Charge 2’s PPG-based HR monitoring in spinal cord injury (SCI) wheelchair users, investigating the impact of exercise intensity and neurological impairment level on measurement accuracy	48 participants (38 with SCI and 10 without)	Polar H7 HR Monitor (chest strap, Bluetooth Low Energy)	Fitbit Charge 2	Overall Accuracy (All Lesions): Mean Absolute Percentage Error (MAPE): 12.99% (outside acceptable ±10% range); Agreement: Moderate (CCC = 0.577). Accuracy by Lesion Level: Non-SCI: 8.09% (within acceptable range); Lesions below T5: 11.16%; Lesions T1–T5: 10.5%; Cervical Lesions (tetraplegia): 20.43% (significantly reduced accuracy); Accuracy by Activity Intensity: Rest: 6.5% (best performance); Moderate Activity: 12.97%; Strength Exercises: 14.2% (worst performance).	HR	Clinical/experimental: rest, wheelchair activities, and a 30 min strength exercise block
Kuo et al. [47]	To assess the feasibility of using imaging PPG (IPPG) from in-vehicle face video for HR monitoring during real-world driving, compared to chest-strap measurements.	10 drivers	Zephyr Bioharness 3.0 (chest strap)	IPPG (camera-based)	48–75% accuracy in 4/10 participants	HR	Occupational (in loco): drivers
Liu et al. [48]	To assess the validity of the Polar Verity Sense (PVS) armband versus the Polar H10 chest strap for HR monitoring during high-intensity interval training (HIIT) in adolescents.	39 students (7th grade)	Polar H10 (chest strap; Bluetooth)	PVS	Strong agreement between PVS and H10 overall (r = 0.93, mean absolute error (MAE) = 4 bpm, 2.8% error). Slightly reduced accuracy at high intensity (≥80% max HR, r= 0.84). Unaffected by sex, body mass index, waist size, or fitness level. PVS is a valid, practical alternative to chest straps for HIIT monitoring in school settings.	HR	Experimental (in lab): HIIT
Milena et al. [49]	To assess the feasibility of deriving HR variability (HRV) metrics from mechanical cardiac signals (recorded via accelerometer and gyroscope) as an alternative to conventional electrical signals (ECG)	22 healthy subjects	Zephyr Bioharness 3.0 (chest strap, 1-lead ECG, 250 Hz)	Inertial Measurement Unit (IMU) sensor (Xsens DOT)	Gyrocardiogram (GCG) in lying posture showed the highest accuracy; seismocardiogram (SCG) was less reliable than GCG, especially in seated posture.	HRV; R-R peaks	Experimental (in lab): sitting (1) and lying (2) posture
Navalta et al. [50]	To determine concurrent heart rate validity during trail running	21 healthy subjects	Polar H7 (chest strap, 1000 Hz)	Garmin Fenix 5 wristwatch, Jabra Elite Sport earbuds, Motiv ring, Scosche Rhythm+ forearm band, Suunto Spartan Sport watch with accompanying chest strap	Garmin Fenix 5 (MAPE = 13%, Bland–Altman Limits of Agreement (LOA) = −32 to 162, Lin’s Concordance Coefficient (rC) = 0.32), Jabra Elite Sport (MAPE = 23%, LOA = −464 to 503, rC = 0.38), Motiv ring (MAPE = 16%, LOA = −52 to 96, rC = 0.29), Scosche Rhythm+ (MAPE = 6%, LOA = −114 to 120, rC = 0.79), Suunto Spartan Sport (MAPE = 2%, LOA = −62 to 61, rC = 0.96).	HR	Experimental (in lab): The trail runs were out and back with the first 1.61 km in an uphill direction, and the 1.61 return being downhill in nature
Navalta et al. [51]	To determine the validity of the PVS optical HR monitor for measuring HR and HRV during rest and exercise, using the Polar H10 chest strap as the criterion device.	17 healthy adults	Polar H10 (chest strap, 1000 Hz, Bluetooth)	PVS (PPG armband)	PVS HR: r = 0.99 vs. H10; HRV: Intraclass Correlation Coefficient = 0.83, r = 0.84; mean bias: −3.3 ms; all within acceptable limits. Wearable, comfortable, good agreement with the criterion device, wrist/arm placement flexibility	HR, HRV	Experimental (in lab): Exercise monitoring
Romano et al. [52]	To compare the performance of accelerometer (ACC) and gyroscope (GYR) sensors (embedded in a single IMU) for simultaneous HR and respiratory rate (RR) monitoring, while evaluating the impact of window length and posture on accuracy	18 healthy subjects	Zephyr Bioharness 3.0 (chest strap, 250 Hz)	Chest-worn IMU (Shimmer3)—Accelerometer and Gyroscope	HR: 5 s windows yielded the worst agreement with ECG, especially in the standing posture (LOA ~ 12.5–12.8 bpm); 55 s windows showed the best agreement (LOA ~ 3.5–3.7 bpm for SCG and GCG); Other window sizes (15–45 s) showed comparable and stable performance. MAE were similar for SCG and GCG, with SCG differing by no more than 0.53 bpm in the seated posture.	HR	Experimental (in lab)

**Table 4:** Chest-strap devices’ technical specifications.

Brand	Polar			Zephyr	Movesense	Garmin	Coospo	Shimmer^®^
Model	H7	H10	T31	Bioharness 3.0	Sensor	HRM-Dual	H6	IFC cnr
**Dimension**	30 × 20 × 9 mm	65 × 34 × 10 mm		28 × 7 mm	36.6 × 10.6 mm	62 × 34 × 11 mm	60 × 33.8 × 12.2	50 × 25 × 23 mm
**Weight**	100 g	60 g		89 g	9.4 g	54.4 g	46.4 g	30 g
**Performance**	No recording mode	CPU velocity: 64 MHz; Memory: MB; Recording mode	Coded; No recording mode	Continuous physiological monitoring; ROG status (Red/Orange/Green) alerts; Data logging up to 500+ h; Multiple transmission modes; Recording mode	Nordic Semiconductor nRF52832, 32-bit ARM Cortex-M4, 64 kB RAM, 512 kB FLASH; Recording mode	No recording mode	No recording mode	12-bit A/D resolution; 200 Hz sampling rate; Recording mode
**Connectivity**	Bluetooth Low Energy (BLE); Analogue; Transmission rate varies with receiving device; Short transmission range (receiving device should be in front of the user, fixed on a belt or pocket)	BLE; Analogue; ANT 2.1; Transmission range: 9000 cm	Analogue 5 kHz	Bluetooth 2.1 + EDR; IEEE 802.15.4 (2.405–2.480 GHz); USB (for charging/configuration)	BLE 4.0/5.0	ANT; BLE 2	Bluetooth (10 m); ANT+ (7 m);	Chipcon CC2420 radio transceiver (2.4 GHz), Rufa™ antenna, RN-41 Bluetooth^®^ module; Short-range transmission (up to 30 m); low-power modes for energy efficiency
**Durability**	−10 °C to +50 °C; Water Resistant (WR)	−10 °C to +50 °C; WR 30 m	WR	IP55 water/dust resistant; Operating Temp: −30 °C to +60 °C; Storage Temp: −40 °C to +85 °C	WR 30 m	WR 10 m	+5 °C a + 40 °C, ≤95% Relativity Humidity	Designed for long-term chest wear; comfortable and adaptable to body shape
**Sensors**	Electrodes	Electrodes; Accelerometer	Electrodes	Electrodes (250 Hz); Respiratory (25 Hz); 3-axis accelerometer (100 Hz); Posture detection; Internal temperature sensor	Accelerometer, Gyroscope, Magnetometer, Temperature, Electrodes	Electrodes	Electrodes	ECG with gain of 175; uses low-power CMOS op-amps; cable-free electrode connection
**Battery**	Type: CR 2025; Lifetime: 200 h; Rechargeable: No; Replaceable: yes	Duration: 165 mAh; Type: coin cell; Rechargeable: No; Replaceable: yes	Duration: 2500 h; Non-replaceable.	Rechargeable Lithium-Polymer (3.7 V); 12–24 h (transmit), 35 h (logging); Charging via USB: 3 h full, 1 h to 90%	CR2025 type battery (lithium coin battery), duration varies depending on use, up to several months	CR2032; 3.5 y (1 h a day); Replaceable: yes; Rechargeable: no	Type: CR2032; 300 h; Replaceable: yes; Rechargeable: no	3 V Li-ion, 280 mAh; transmission: 60 mA; reception: 40 mA; idle: 1.4 mA; sleep: 50 µA
**Extra features**	Discontinued from 2020	Firmware upgradeable		ROG status logic configurable; Software Development Kit available; Data export in CSV/HEX for analysis (e.g., MATLAB)	Software controllable red LEDs, 3 Mbit EEPROM for storage, API for development, OTA firmware			Includes 2 GB SD Card for onboard storage; SPI via USART1; suitable for wearable applications

**Table 5:** General comparison of included chest-strap devices.

Sensor (Model)	Key Advantages	Key Limitations	Application-Specific Notes/Trade-offs
**Polar H7**	High correlation with ECG (r ≈ 0.99); high sampling rate (≥1000 Hz); widely used as reference; Bluetooth connectivity; low cost; real-time data transmission; usable in children	Susceptible to motion artefacts; limited raw data; not FDA/CE approved	Good for low- to moderate-intensity lab or clinical settings; less reliable during high-intensity or dynamic activity
**Polar H10**	User-friendly; stable electrode contact; high sampling rate (≥1000 Hz); Bluetooth connectivity; affordable; reliable for HRV measures; usable in children; excellent RR interval accuracy	Strap placement sensitivity; discomfort in long-term wear; limited raw data; not CE approved	Strong choice for lab, sports, and clinical research requiring HRV; trade-off between comfort and precision during long sessions
**Polar T31**	Low cost; lightweight; user-friendly; water-resistant	Lacks advanced HRV/ECG; less accurate at high intensity	Suitable for general exercise and fitness monitoring; not recommended for research or clinical HRV assessment
**Zephyr BioHarness 3.0**	Multimodal (ECG, HR, respiration, accelerometry); Bluetooth connectivity; field-based; comfortable with low skin irritations; long battery; accurate RR interval; supports cardiac–locomotor entrainment	Bulkier; reduced comfort for long-term wear; signal degradation with sweat	Excellent for field-based occupational or sports monitoring; trade-off between multimodal functionality and comfort; very suitable for stress and physiological studies
**Movesense HR+ / Medical / ECG**	High accuracy (MAPE <1%); Bluetooth connectivity; real-time ECG/HRV transmission; user-friendly	Limited population validation; less accurate at high intensity; strap discomfort when long-term wear	Ideal for research or monitoring intense activity; less validated in children/older adults
**Garmin HRM-Dual**	Affordable; reliable HR during moderate exercise; good agreement with other straps	Less accurate HRV; weaker under in-motion activity	Best for moderate exercise; limited HRV precision; comfort may be preferred over advanced metrics
**Coospo H6**	Low cost; good HR accuracy	Limited validation; less stable in high-intensity activity	Suitable for general fitness; not ideal for high-intensity sports or HRV research
**Shimmer^®^ IFC-CNR**	Research-grade ECG; comfortable; customizable; Bluetooth connectivity; allows real-time and offline analysis; usable in children; experimental studies	Expensive; less user-friendly; limited general availability	Best for controlled experimental settings; not practical for field or consumer use

**Table 6:** Quality assessment of studies evaluating chest-strap devices.

Reference	SD	S1	S2	SD.1	SD.2	SD.3	SD.4	SD.5	Level
Bläsing et al. [21]	3	Y	Y	N	Y	Y	CT	Y	Medium
Constantini et al. [22]	3	Y	Y	Y	Y	Y	CT	Y	High
Etiwy et al. [23]	4	Y	Y	Y	Y	Y	Y	Y	High
Flores et al. [24]	3	Y	Y	N	Y	Y	N	CT	Medium
Gilgen-Ammann, Schweizer, and Wyss [25]	4	Y	Y	Y	N	Y	Y	Y	High
Martín Gómez et al. [26]	4	Y	Y	Y	N	Y	Y	Y	High
Kuo et al. [27]	3	Y	Y	Y	Y	Y	Y	Y	High
Marzano-Felisatti et al. [28]	3	Y	Y	N	Y	Y	CT	Y	Medium
Maza, Goizueta, and Llorens [29]	3	Y	Y	N	Y	Y	CT	Y	Medium
Mishra et al. [30]	3	Y	Y	N	Y	Y	CT	Y	Medium
Montes and Navalta [31]	3	Y	Y	N	Y	Y	CT	Y	Medium
Nuske et al. [32]	3	Y	Y	Y	Y	Y	Y	Y	High
Di Palma et al. [33]	3	Y	Y	CT	Y	Y	N	Y	Medium
Parak et al. [34]	3	Y	Y	CT	Y	Y	Y	Y	High
Plews et al. [35]	3	Y	Y	CT	Y	Y	N	Y	Medium
Rogers et al. [36]	3	Y	Y	CT	Y	N	N	Y	Low
Romagnoli et al. [37]	4	Y	Y	Y	N	Y	Y	Y	High
Saggu et al. [38]	3	Y	Y	CT	Y	N	CT	Y	Low
Skála et al. [54]	3	Y	Y	Y	Y	Y	Y	Y	High
Speer et al. [40]	3	Y	Y	Y	Y	Y	Y	CT	High
Van Oost et al. [41]	3	Y	Y	N	Y	Y	CT	Y	Medium
Vila et al. [42]	3	Y	Y	N	Y	CT	CT	Y	Low

Legend: SD: Study Design (1—qualitative studies; 2—quantitative randomized controlled trials; 3—quantitative non-randomized studies; 4—quantitative descriptive studies; 5—mixed methods studies). S1. Are there clear research questions? S2. Do the collected data allow for addressing the research questions? Questions: 3.1. Are the participants representative of the target population? 3.2. Are measurements appropriate regarding both the outcome and intervention (or exposure)? 3.3. Are there complete outcome data? 3.4. Are the confounders accounted for in the design and analysis? 3.5. During the study period, was the intervention administered (or exposure occurred) as intended? 4.1. Is the sampling strategy relevant to address the research question? 4.2. Is the sample representative of the target population? 4.3. Are the measurements appropriate? 4.4. Is the risk of nonresponse bias low? 4.5. Is the statistical analysis appropriate to answer the research question? Y—Yes; N—No; CT—Can’t tell. Quality rating: high: 0 or 1 N or CT; medium: 2 N or CT; low: 3 or more N or CT.

**Table 7:** Quality assessment of studies using chest-strap devices as reference.

Reference	SD	S1	S2	SD.1	SD.2	SD.3	SD.4	SD.5	Level
Chow and Yang [43]	3	Y	Y	Y	Y	Y	CT	Y	High
Cosoli et al. [44]	3	Y	Y	N	Y	Y	CT	Y	Medium
Higgins et al. [45]	3	Y	Y	N	Y	N	CT	Y	Low
Hoevenaars et al. [46]	3	Y	Y	Y	Y	Y	Y	Y	High
Kuo et al. [47]	3	Y	Y	N	Y	Y	Y	Y	High
Liu et al. [48]	3	Y	Y	Y	Y	Y	Y	Y	High
Milena et al. [49]	3	Y	Y	N	Y	Y	CT	Y	Medium
Navalta et al. [50]	3	Y	Y	CT	Y	Y	N	Y	Medium
Navalta et al. [51]	3	Y	Y	CT	Y	Y	CT	Y	Medium
Romano et al. [52]	3	Y	Y	N	Y	Y	CT	Y	Medium

Legend: SD—Study Design (1—qualitative studies; 2—quantitative randomized controlled trials; 3—quantitative non-randomized studies; 4—quantitative descriptive studies; 5—mixed methods studies) S1. Are there clear research questions? S2. Do the collected data allow for addressing the research questions? Questions: 3.1. Are the participants representative of the target population? 3.2. Are measurements appropriate regarding both the outcome and intervention (or exposure)? 3.3. Are there complete outcome data? 3.4. Are the confounders accounted for in the design and analysis? 3.5. During the study period, was the intervention administered (or exposure occurred) as intended? Y—Yes; N—No; CT—Can’t tell. Quality rating: high: 0 or 1 N or CT; medium: 2 N or CT; low: 3 or more N or CT.

## Data Availability

Not applicable.

## Abbreviations

The following abbreviations are used in this manuscript:

BMI	Body Mass Index
CE	*Conformité Européenne*
ECG	Electrocardiogram
FDA	Food and Drug Administration
HR	Heart Rate
HRV	Heart Rate Variability
IBI	Inter-Beat Interval
IMU	Inertial Measurement Unit
LOAs	Reported Limits of Agreement
MAPE	Absolute Mean Errors Below 1%
MMAT	Mixed Methods Appraisal Tool
OSH	Occupational Safety and Health
PPE	Personal Protective Equipment
PPG	Photoplethysmography
RRIs	RR Intervals

## Appendix A. Comparative Table Summarizing Recent Reviews on Wearable Cardiac Monitoring and Explicitly Highlighting Its Contributions

**Article**	**Device Focus**	**Systematic Method (PRISMA)**	**Contexts Analyzed**	**Value Added**
**Vermunicht et al., 2025 [15]**	Chest-worn ECG strap	Yes	Cardiac patients in clinical care	Exclusive validation on cardiac patients; 24h monitoring outside of exercise; focus on ECG chest straps
**Jamieson et al., 2025 [14]**	Consumer-grade wearables	No - Guide	General health/cardiovascular	Broad device comparison (wrist, chest, patch, etc.); lacks systematic context focus
**Murray et al., 2025 [18]**	General wearables	Yes	Heart failure & pulmonary congestion	Emphasis on clinical diagnostics; sensor type not exclusive
**Ranjan et al., 2025 [17]**	Multiple wearable types	Yes	Clinical, ambulatory, telehealth	Analysis of ECG/PPG in diverse devices; not chest-focused
**Wang et al., 2024 [19]**	Consumer ECG wearables	Yes	Cardiac health monitoring	Focuses on consumer devices and ECG-diagnosing algorithms; strong emphasis on deep learning/CNNs for ECG analysis
**Dahiya et al., 2024 [16]**	Chest, wrist, patch, textile	No - Scoping review	Healthy adults, cardiac patients	Summarizes 12 studies; compares device types (including chest straps, textile shirts), safety, accuracy, minor adverse effects

## Appendix B. Search Strings and Corresponding Results by Database

**Database**	**Query**	**Results**
Pubmed	("Chest Strap" OR "Thoracic Sensor" OR "Wearable Sensor" OR "Wearable devices" OR "Wearable" OR "Chest-worn" OR "Thoracic band" OR "Chest belt" OR " Chest monitor")	39,048
	("Physiologic* Data" OR "Heart Rate" OR “HR” OR "Biometric" OR "Heart Rate Monitoring" OR "Biometry" OR "Blood Pressure" OR “Physiologic* signals" OR “Physiological measures” OR “Physiological Parameters” OR “Physiologic* Monitoring" OR "Ambulatory Monitoring" OR "Ambulatory Blood Pressure Monitoring" OR "Ambulatory Electrocardiography" OR "ECG" OR "Ambulatory Electrocardiography Monitoring" OR “Electrocardiogram” OR “Heart Rate Variability” OR “HRV” OR “Cardiac* stress”)	1,259,126
	#1 AND #2	9064
	Plus filters	682
Science Direct	(“Chest Based Sensor” OR “Chest Strap” OR “Wearable”) AND (“Heart Rate” OR “Heart Rate Variability” OR “Cardiac Stress” OR “Electrocardiogram”)	34,704
	Plus Filters	969
Web of Science	TS= ("Chest Strap" OR "Thoracic Sensor" OR "Wearable Sensor" OR "Wearable devices" OR "Wearable" OR "Chest-worn" OR "Thoracic band" OR "Chest belt" OR " Chest monitor") https://www.webofscience.com/wos/woscc/summary/b927efd4-0e7f-4c16-bd1c-8c58a3149b3c-0162417e45/relevance/1 (accessed on 23 September 2025).	98,757
	TS= ("Physiologic* Data" OR "Heart Rate" OR “HR” OR "Biometric" OR "Heart Rate Monitoring" OR "Biometry" OR "Blood Pressure" OR “Physiologic* signals" OR “Physiological measures” OR “Physiological Parameters” OR “Physiologic* Monitoring" OR "Ambulatory Monitoring" OR "Ambulatory Blood Pressure Monitoring" OR "Ambulatory Electrocardiography" OR "ECG" OR "Ambulatory Electrocardiography Monitoring" OR “Electrocardiogram” OR “Heart Rate Variability” OR “HRV” OR “Cardiac* stress”) https://www.webofscience.com/wos/woscc/summary/8e6ff41b-268f-4dd3-9a5d-1e993fff617e-0162418751/relevance/1 (accessed on 23 September 2025).	1,127,002
	#1 AND #2 (https://www.webofscience.com/wos/woscc/summary/65cf83c4-e063-4d96-b9b4-3f59d5664cf1-016241a15a/relevance/1 (accessed on 23 September 2025))	13,318
	Plus filters (https://www.webofscience.com/wos/woscc/summary/9beed659-2175-48c7-9945-7b63aac1448d-016242107d/relevance/1 (accessed on 23 September 2025))	4298
Scopus	TITLE-ABS-KEY ("Chest Strap" OR "Thoracic Sensor" OR "Wearable Sensor" OR "Wearable devices" OR "Wearable" OR "Chest-worn" OR "Thoracic band" OR "Chest belt" OR " Chest monitor") AND TITLE-ABS-KEY ("Physiologic* Data" OR "Heart Rate" OR “HR” OR "Biometric" OR "Heart Rate Monitoring" OR "Biometry" OR "Blood Pressure" OR “Physiologic* signals" OR “Physiological measures” OR “Physiological Parameters” OR “Physiologic* Monitoring" OR "Ambulatory Monitoring" OR "Ambulatory Blood Pressure Monitoring" OR "Ambulatory Electrocardiography" OR "ECG" OR "Ambulatory Electrocardiography Monitoring" OR “Electrocardiogram” OR “Heart Rate Variability” OR “HRV” OR “Cardiac* stress”)	21,288
	Plus Filters	5926
